# A statistical approach for array CGH data analysis

**DOI:** 10.1186/1471-2105-6-27

**Published:** 2005-02-11

**Authors:** Franck Picard, Stephane Robin, Marc Lavielle, Christian Vaisse, Jean-Jacques Daudin

**Affiliations:** 1 Institut National Agronomique Paris-Grignon, UMR INAPG/ENGREF/INRA MIA 518, Paris, France; 2 Université Paris Sud, Equipe Probabilités, Statistique et Modélisation, Orsay, France; 3 University of California San Francisco, Diabetes Center, San Francisco, USA

## Abstract

**Background:**

Microarray-CGH experiments are used to detect and map chromosomal imbalances, by hybridizing targets of genomic DNA from a test and a reference sample to sequences immobilized on a slide. These probes are genomic DNA sequences (BACs) that are mapped on the genome. The signal has a spatial coherence that can be handled by specific statistical tools. Segmentation methods seem to be a natural framework for this purpose. A CGH profile can be viewed as a succession of segments that represent homogeneous regions in the genome whose BACs share the same relative copy number on average. We model a CGH profile by a random Gaussian process whose distribution parameters are affected by abrupt changes at unknown coordinates. Two major problems arise : to determine which parameters are affected by the abrupt changes (the mean and the variance, or the mean only), and the selection of the number of segments in the profile.

**Results:**

We demonstrate that existing methods for estimating the number of segments are not well adapted in the case of array CGH data, and we propose an adaptive criterion that detects previously mapped chromosomal aberrations. The performances of this method are discussed based on simulations and publicly available data sets. Then we discuss the choice of modeling for array CGH data and show that the model with a homogeneous variance is adapted to this context.

**Conclusions:**

Array CGH data analysis is an emerging field that needs appropriate statistical tools. Process segmentation and model selection provide a theoretical framework that allows precise biological interpretations. Adaptive methods for model selection give promising results concerning the estimation of the number of altered regions on the genome.

## Background

Chromosomal aberrations often occur in solid tumors: tumor suppressor genes may be inactivated by physical deletion, and oncogenes activated via duplication in the genome. Gene dosage effect has become particularly important in the understanding of human solid tumor genesis and progression, and has also been associated with other diseases such as mental retardation [1,2]. Chromosomal aberrations can be studied using many different techniques, such as Comparative Genomic Hybridization (CGH), Fluorescence in Situ Hybridization (FISH), and Representational Difference Analysis (RDA). Although chromosome CGH has become a standard method for cytogenetic studies, technical limitations restrict its usefulness as a comprehensive screening tool [3]. Recently, the resolution of Comparative Genomic Hybridizations has been greatly improved using microarray technology [4,5].

The purpose of array-based Comparative Genomic Hybridization (array CGH) is to detect and map chromosomal aberrations, on a genomic scale, in a single experiment. Since chromosomal copy numbers can not be measured directly, two samples of genomic DNA (referred to as the reference and test DNAs) are differentially labelled with fluorescent dyes and competitively hybridized to known mapped sequences (referred to as BACs) that are immobilized on a slide. Subsequently, the ratio of the intensities of the two fluorochromes is computed and a CGH profile is constituted for each chromosome when the log_2 _of fluorescence ratios are ranked and plotted according to the physical position of their corresponding BACs on the genome [6]. Different methods and packages have been proposed for the visualization of array CGH data [7,8].

Each profile can be viewed as a succession of "segments" that represent homogeneous regions in the genome whose BACs share the same relative copy number on average. Array CGH data are normalized with a median set to log_2_(ratio) = 0 for regions of no change, segments with positive means represent duplicated regions in the test sample genome, and segments with negative means represent deleted regions. Even if the underlying biological process is discrete (counting of relative copy numbers of DNA sequences), the signal under study is viewed as being continuous, because the quantification is based on fluorescence measurements, and because the possible values for chromosomal copy numbers in the test sample may vary considerably, especially in the case of clinical tumor samples that present mixtures of tissues of different natures.

Two main statistical approches have been considered for the analysis of array CGH data. The first has focused many attentions, and is based on segmentation methods where the purpose is to locate segments of biological interest [7,9-11]. A second approach is based on Hidden Markov Models (aCGH R-package [12]), where the purpose is to cluster individual data points into a finite number of hidden groups. Our approach can be put into the first category. Segmentation methods seem to be a natural framework to handle the spatial coherence of the data on the genome that is specific to array CGH. In this context the signal provided by array CGH data is supposed to be a realization of a Gaussian process whose parameters are affected by an unknown number of abrupt changes at unknown locations on the genome. Two models can be considered, according to the characteristics of the signal that is affected by the changes: it can be either the mean of the signal [7,10,11] or the mean and the variance [9]. Since the choice of modeling is crucial in any interpretation of a segmented CGH profile, we provide guidelines for this choice in the discussion. Two major issues arise in break-points detection studies: the localization of the segments on the genome, and the estimation of the number of segments. The first point has lead to the definition of many algorithms and packages: segmentation algorithms [9,10] and smoothing algorithms [11] where the break-points are defined with a *posterior *empirical criterion. These methods are defined by a criterion to optimize and an algorithm of optimization. Different criteria have been proposed: the likelihood criterion [9,11], the least-squares criterion [7], partial sums [10], and algorithms of optimization are based on genetic algorithms [9], dynamic programing [7], binary segmentation (DNAcopy R-package [10]) and adaptive weigths smoothing (GLAD R-package [11]). Since many criteria and algorithms have been proposed, one important question is the resulting statistical properties of the break-point estimators they provide. Note that smoothing techniques do not provide estimators of the break-point coordinates, since the primary goal of the underlying model is to smooth the data, and break-points are not parameters of the model (in this case, they are defined after the optimization of the criterion [11]). Here we consider the likelihood criterion and we use dynamic programming that provides a global optimum solution, contrary to genetic algorithms [9], in a reasonable computational time.

As for the estimation of the number of segments, the existing articles have not defined any statistical criterion adapted to the case of process segmentation. This problem is theoretically complex, and has lead to *ad hoc *procedures [9-11]. Since the purpose of array CGH experiments is to discover biological events, the estimation of the number of segments remains central. This problem can be handled in the more general context of model selection. In the discussion we explain why classical criteria based on penalized likelihoods are not valid for break-points detection. Criteria such as the Akaike Information Criterion (AIC) and the Bayes Information Criterion (BIC) lead to an overestimation of the number of segments. For this reason, an arbitrary penalty constant can be chosen in order to select a lower number of segments in the profile [9]. We propose a new procedure to estimate the number of segments, choosing the penalty constant adaptively to the data. We explain the construction of such penalty, and its performances are compared to other criteria in the Results Section, based on simulation studies and on publicly available data sets. Put together, we propose a methodology that considers a simple modeling, a fast and effective algorithm of optimization and that takes advantages of the statistical properties of the maximum likelihood. Our procedure has been implemented on MATLAB Software and is freely available .

## Results

### Comparison of model selection criteria

**Figure 1:**
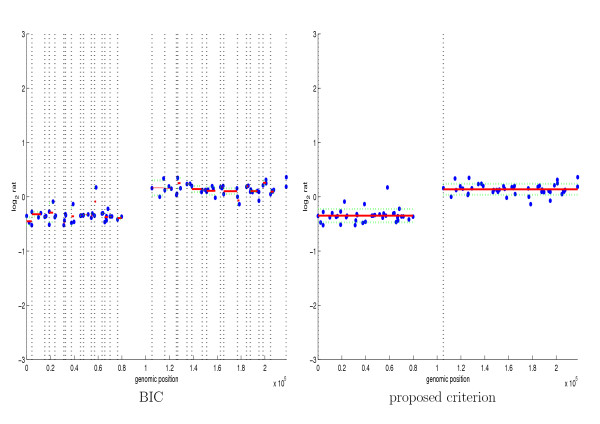
**Results of the segmentation procedure when using the Bayesian Information Criterion (BIC) and the proposed criterion. **Data shown corresponds to Coriell cell lines GM03563, chromosome 3. Red lines represent the estimated mean of each segments, and green lines, the estimated mean plus one standard deviation.

To show the importance of the choice of the model selection criterion on simple data, we use the results of a single experiment performed on fibroblast cell lines (see the Materials Section), with one known chromosomal aberration. Figure 1 shows the resulting segmentations when using the Bayesian Information Criterion, and our criterion. BIC leads to an oversegmented profile that is not interpretable in terms of relative copy numbers. Our procedure estimates the correct number of segments 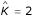. This example shows the practical consequences of the use of theoretically unappropriated criteria. This point constitutes the main purpose of the discussion (see the Discussion Section).

**Figure 2:**
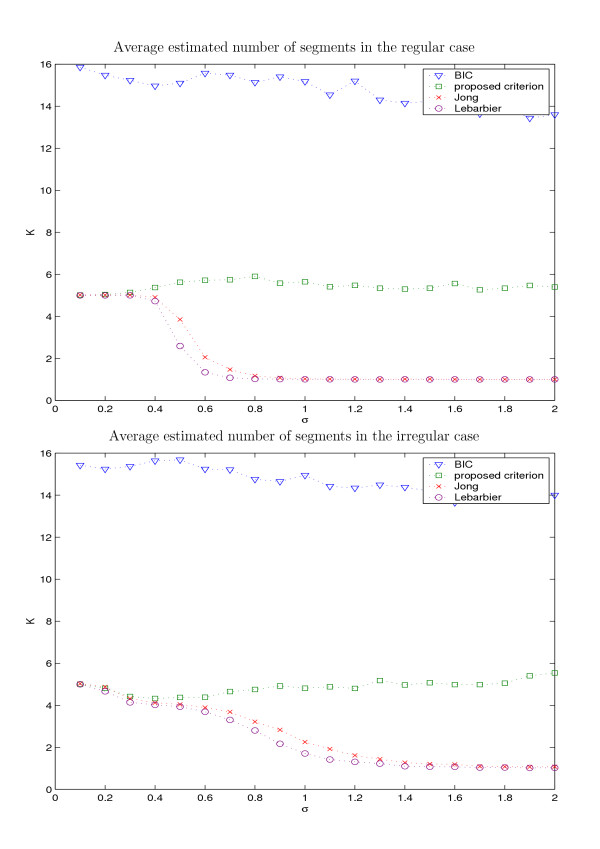
**Estimated number of segments for 4 different penalized criteria in the regular case (top) and the irregular case (bottom). **Top : Results of the simulations for 5 regularly spaced segments with n = 100 data points. The graph represents the average estimated number of segments for each criterion according to the standard deviation of the noise (*σ*). Bottom: Results of the simulations for 5 unregularly spaced segments with *n *= 100 data points. The adaptive criterion is robust to the additional noise since it maintains an estimate close to 5 segments whatever the noise and the configuration.

**Figure 3:**
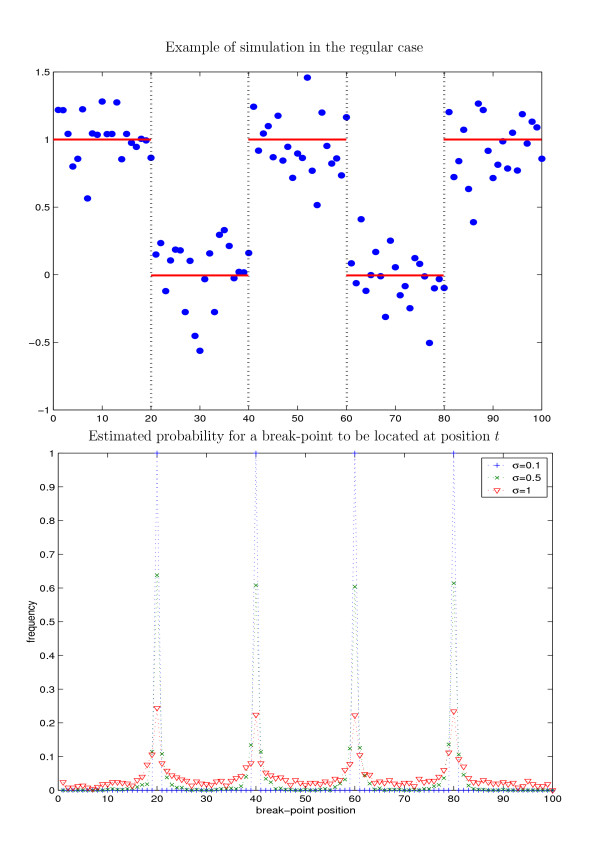
**Example of a simulation in the regular case, and result of the dynamic programming algorithm for the estimation of the break-point coordinates. **Top: Example of simulation for 100 data points and 5 segments in the regular case. The true break-points are designated by vertical lines, and the red lines correspond to the mean of each segment. The difference of means *d *is constant and equals 1. Bottom: Estimated frequency for a break-point to be located at coordinate *t *for *t *= 1 to 100. Different levels of noise are considered with *σ *= 0.1, *σ *= 0.5, *σ *= 1.

**Figure 4:**
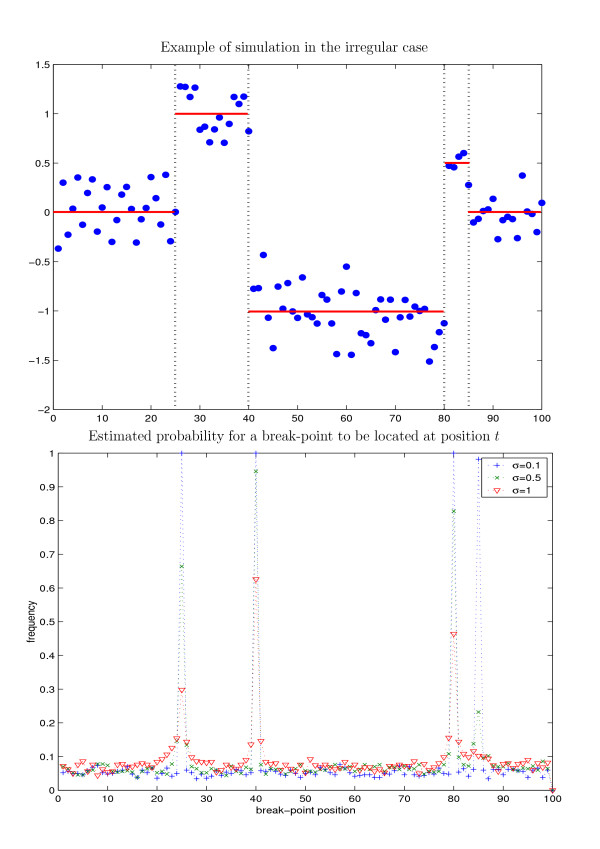
**Example of a simulation in the irregular case, and result of the dynamic programming algorithm for the estimation of the break-point coordinates. **Top: Example of simulation for 100 data points and 5 segments in the irregular case. The true break-points are designated by vertical lines, and the red lines correspond to the mean of each segment. The difference of means varies between *d *= 2 to *d *= 0.5. Bottom: Estimated probability for a break-point to be located at coordinate *t *for *t *= 1 to 100. Different levels of noise are considered with *σ *= 0.1, *σ *= 0.5, *σ *= 1.

Numerical simulations are performed to study the sensitivity of different criteria to varying amounts of noise. The simulation design is described in the Methods Section. We compare four different criteria: the Bayesian Information Criterion, two previously described criteria [9,13], and the criterion we propose, in their ability to estimate the correct number of segments. Two configurations were tested, for a true number of segments K* = 5. In the first situation, the segments are regularly spaced with a jump of the mean of 1 (Figure 3), whereas in the second case, the segments are not regularly spaced and the differences of means vary between *d *= 2 and *d *= 0.5 (Figure 4). The first result is that BIC overestimates the number of segments, whatever the noise and the configuration (Figure 2). On the contrary, previously described criteria [9,13] tend to underestimate the number of segments when the noise increases, whatever the configuration. These results suggest that those two criteria "prefer" to detect no break-point as the noise increases, leading to possible false negative results.

The behavior of the criterion we propose is different. It seems to be more robust to the noise, as it will give a number of segments that is close to the true number. In particular, the irregular configuration presents a segment of small size (5 points at *t *= 80) that could be interesting to detect in the case of array CGH profile (a putative gained region for instance). Since the previously described criteria [9,13] tend to underestimate the number of segments, this particular region would not be detected. On the contrary, the adaptive criterion will be able to detect it, even if the noise is important, since it selects a constant number of segments close to the true number whatever the noise. These simulation examples perfectly illustrate the capacity of an adaptive criterion to find a reasonable number of segments even in configurations where the profile is not very separated.

**Figure 5:**
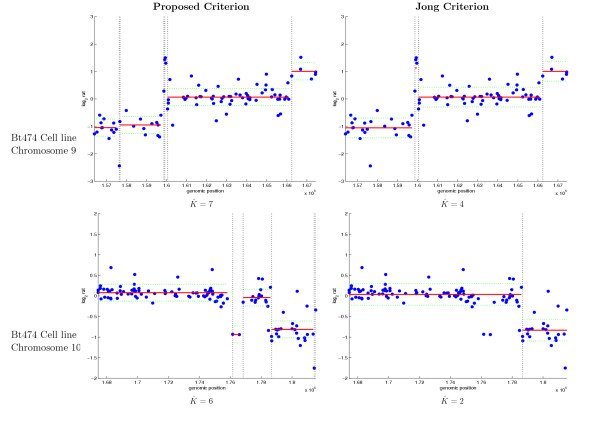
**Comparison of segmentation results based on Breast Cancer Cell lines using the adaptive criterion and Jong criterion. **Results of the segmentation procedure for Breast cancer cell lines Bt474, chromosomes 9 and 10. Fluoresence log_2_-ratios are plotted according to their location on the genome in megabases. Left profiles are segmented using the adaptive criterion and right profiles using Jong's criterion. The adaptive method detects a break-point at 1.58 MB on chromosome 9 that seems to be an outlier, and detects a putative deleted region on chromosome 10 at 1.76 MB.

We also compare the performance of our criterion and of the arbitrary criterion [9] on breast cancer cell lines. Figure 5 shows the resulting segmentations on chromosomes 9 and 10 of the Bt474 cell line (see the Materials Section for further description). As previously mentioned, the arbitrary criterion [9] selects a lower number of segments compared to the adaptive criterion, and we note that interesting regions are not detected (a putative outlier on chromosome 9 at 1.58 Mb and a putative deleted region on chromosome 10 at 1.76 Mb). Since the aim of array CGH experiments is to discover unknown chromosomal aberrations, the use of an adaptive criterion seems more appropriate in this context since it allows the identification of regions that seem biologically relevent.

The second simulation-based result concerns the ability of dynamic programming to locate the break-points at the correct coordinate, given different amounts of noise (Figures 3 and 4). In the regular configuration (Figure 3), simulation results show that dynamic programming perfectly localizes the break-points when the variability of the noise *σ*^2 ^is low regarding the jump *d *of the mean. If *d*/*σ *= 10 the estimated probability to localize the break-points at the correct coordinate is 1, and this probability deacreases with the noise (probability close to 0.65 for *d*/*σ *= 2 and 0.25 for *d*/*σ *= 1). The effect of additional noise is to widden the zone of estimation, but the estimated break-points remain close to the true break-points. If the true break-point is located at t*, the estimated break-point stays in the interval t* ± 3. In the irregular configuration, additional noise has similar effects on the break-point's positioning, but the probability to correctly estimate a break-point depends on the jump of the mean between two segments. In the irregular case, Figure 4, at position *t *= 40 the difference of mean is *d *= 2, and the probability to locate the break-point at the true coordinate is higher than 0.65 for any additional noise. On the contrary, at position *t *= 85 where the different of mean equals *d *= 0.5 the probability to correctly locate the break-point decreases dramatically with the noise (probability 1 for *σ *= 0.1 and probability 0.25 for *σ *= 0.5). This means that dynamic programming is sensitive to small segments that present little differences in the mean regarding the noise. Nevertheless, the example on the real data set presented in Figure 5 shows that using an adaptive criterion with dynamic programming allows for the identification of small regions of putative biological interest as mentioned above. Put together, these simulation results show that the adaptive method selects the good number of segments even in the presence of important noise, and that when this number is selected, dynamic programming is able to correctly localize the break-point. In addition to its ability to locate precisely the break-points, it is important to notice that dynamic programming provides a global optimum of the likelihood that is required for any model selection procedure to select the number of segments, compared to genetic algorithms [9].

### Segmentation models in the Gaussian framework

The CGH profile is supposed to be a Gaussian signal. In a segmentation framework, two types of changes can be considered: changes in the mean and the variance of the signal, or changes in the mean only. Let us define model 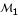 where each segment has a specific mean and variance [9], and model 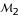, where the variance is common between segments [7].

**Figure 6:**
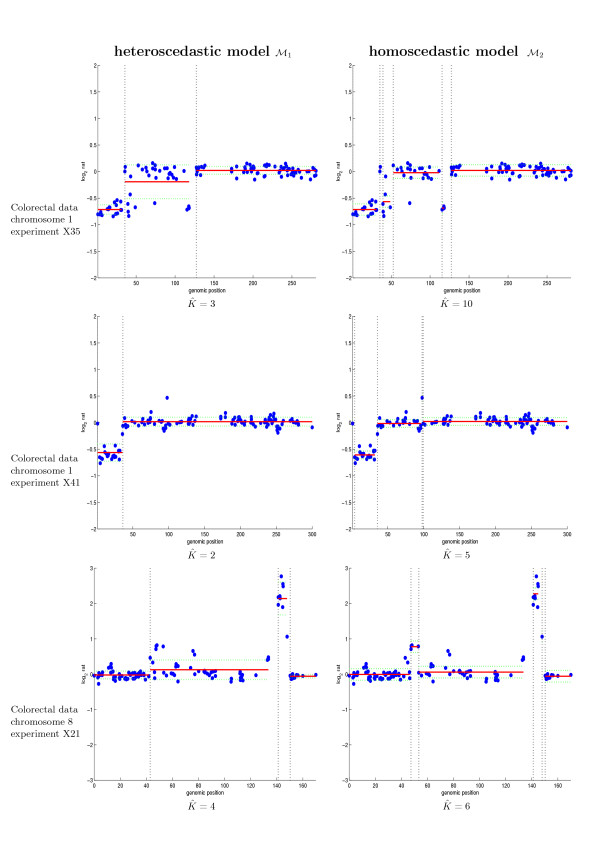
**Comparison of segmentation results based on colorectal cancer data, using model **
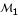
**and **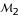. Results of the segmentation procedure for colorectal cancer data, chromosome 1 and chromosome 8. Fluoresence log_2_-ratios are plotted according to their location on the genome in megabases. Left profiles are segmented using model 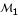, and right profiles using model 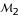. Our criterion is used to estimate the number of segments.

Since both models can be used, it is important to explore their behavior in order to know which model is the best adapted to the special case of array CGH data. We use clinical data obtained from primary dissected tumors of colorectal cancers (see the Materials Section for further details). Figure 6 presents the results of segmentations for three experiments obtained with the two models 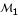 and 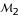 when our criterion is used to estimate the number of segments. The main result of this comparison is that the number of segments is higher using model 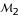 compared to model 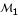. This behavior of model 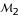 could be interpreted as a trend to divide large segments into smaller parts, in order to maintain the variance homogeneous between segments. This leads to a more segmented profile, maybe more precise, but that may be more difficult to interpret in terms of relative copy numbers. Nevertheless, as model 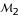 allows the exploration of segments with one observation, it will be more efficient for the identification of outliers, as shown in Figure 6 (experiment X411, model 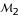, point at 100 Mb).

## Discussion

The definition of an appropriate penalized criterion has been an issue for previous works using segmentation methods for array CGH data analysis [8,9,11]. In this section, we explain the specificity of model selection in the case of process segmentation, in order to give further justification to the inefficiency of classical criteria to select the number of segments, as shown in the Results Section.

### Estimating the number of segments via penalized likelihood

When the number of segments is known, the maximization of the log-likelihood 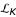 gives the best segmentation with *K *segments (see the Methods Section). In real situations this number is unknown, and one has to choose among many possible segmentations. The maximum of the log-likelihood 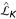 can be viewed as a quality measurement of the fit to the data of the model with *K *segments, and will be maximal when each data point is in its own segment. Therefore selecting the number of segments only based on the likelihood criterion would lead to overfitting. Furthermore, the number of parameters to estimate is proportional to the number of segments, and a too large number of segments would lead to a large estimation error. A penalized version of the likelihood is used as a trade-off between a good adjustement and a reasonable number of parameters to estimate. It is noted


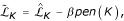


where *pen*(*K*) is a penalty function that increases with the number of segments, and *β *is a constant of penalization. The estimated number of segments is such as :


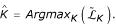


It is crucial to notice that the criterion which is penalized should provide the best partition of *K*-dimensional, *ie *for a fixed *K *the criterion has to be globally maximized to ensure convergence of the break-point estimators to the true break-points [14]. This optimum is provided by dynamic programming, but not by other algorithms [9,10].

### Choice of the penalty function and constant

Classical penalized likelihoods use the number of independent continuous parameters to be estimated as a penalty function. Even though those criteria are widely used in the context of model selection, theoretical considerations suggest that they are not appropriate in the context of an exhaustive search for abrupt changes.

Let us focus on the penalty function in a first step. Table 1 provides a summary of different penalties. For classical information criteria, such as the Akaike Information Criterion and the Bayes Information Criterion, the penalty function equals to 2*K *(*K *means and *K *variances) for a heteroscedastic model with *K *segments. Penalized criteria have already been used in the context of array CGH data analysis to estimate the number of segments [9]. In addition to the 2*K *parameters, they implicitly consider that the break-points are also continuous parameters, leading to a new penalty function *pen*(*K*) = 3*K *- 1, which considers *K *- 1 break-points. Nevertheless, the characteristic of break-point detection models lies in the mixture of continuous parameters and discrete parameters that can not be counted as continuous parameters, since the number of possible configurations for *K *segments is finite and equals 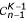 (with *n *the total number of points) [13].

This leads to the definition of a new penalty function adapted to the special context of the exhaustive search of abrupt changes. This function (table 1) is proportional to the number of continuous parameters, but is also proportional to a new term in 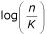 that takes the complexity of the visited configurations into account. It is written *pen*(*K*) = 2*K*(*c*_1 _+ *c*_2_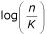), where *c*_1 _and *c*_2 _are constant coefficients that have to be calibrated using numerical simulations. Since AIC and BIC and the criterion proposed in [9] do not consider the complexity of the visited models, they select a too high number of segments. The second term of the penalty is the penalty constant *β*. This term is constant in the case of AIC and BIC (*β *= 1, *β *= 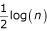, respectively), and contributes to the oversegmentation as mentioned above. This can lead to an empirical choice for the constant, in order to obtain expected results based on *a priori *knowledge. For this reason, an arbitrary penalty constant can be chosen for the procedure to select a reasonable number of segments (*β *= 10/3 in [9]). Instead of an arbitrary choice for this constant, *β *can be adaptively chosen to the data [13,14]. Furthermore, when the number of segments is small with respect to the number of data points (which is the case in CGH data analysis), the log-term can be considered as a constant [14]. The author rather suggests to use the penalty function *pen*(*K*) = 2*K *and to define an automatic procedure to choose the constant of penalization *β *adaptively. We explain the estimation procedure for the penalty constant in the Methods Section.

The power of adaptive methods for model selection lies in the definition of a penalty that is not universal (such as in the case of AIC and BIC). This means that the dimension of the model is estimated adaptively to the data. The efficiency of such method has been shown on simulated data as well as on experimental results (Results Section), and adaptive model selection criteria seem to be very appropriate for array CGH data analysis.

### Choice of modelling for array CGH data

Since the choice of modeling affects the resulting segmentation, it is crucial to provide guidelines for their use. This can be done with the interpretation of the statistical models in terms of their biological meaning. The difference between model 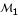 and 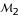 concerns the modeling of the variance: model 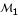 assumes that the variability of the signal is organized along the chromosome, whereas model 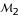 specifies that the variance is constant. Since it has been shown that the vast majority of clones all had the same response to copy number changes in the aneuploid cell lines [6], the use of model 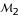 would be justified regarding this experimental argument.

Outliers seem to be a major concern in microarray CGH data analysis. For instance, if only one BAC is altered whereas its neighbors are not, the conclusion could be either that it is biologically relevant, or that the signal is due to technical artefacts. Replications are crucial in this situation, as well as secondary validations. An other possibility could be that the BAC is misannotated: if the ratio is plotted at the wrong coordinate on the genome, it will appear as an outlier, when it is not. The importance of outlier identification is another argument in favor of model 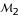, that can detect changes for one data point, whereas with model 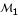 outliers would belong to segments with higher variance.

It has to be noted that classical models used in segmentation methods assume the independence of the data. This may be a reasonnable assumption for BAC arrays whose genome representation is approximately 1 BAC every 1.4 Mb [6]. Nevertheless, a new generation of arrays now provides a tiling resolution of the genome [15]. The overlapping of successive BACs could lead to statistical correlations that will require developments of new segmentation models for correlated processes.

## Conclusions

Microarray CGH currently constitutes the most powerful method to detect gain or loss of genetic material on a genomic scale. To date, applications have been mainly restricted to cancer research, but the emerging potentialities of this technique have also been applied to the study of congenital and acquired diseases. As expression profile experiments require careful statistical analysis before any biological expertise, CGH microarray experiments will require specific statistical tools to handle experimental variability, and to consider the specificity of the the studied biological phenomena. We introduced a statistical method for the analysis of CGH microarray data that models the abrupt changes in the relative copy number ratio between a test DNA and a reference DNA. We discuss the effects of different modelings that can be used in segmentation methods, and suggest the use of a model that considers the homogeneity of the signal variability based on experimental arguments and regarding the specificity of array CGH data.

The main theoretical issue of array CGH data analysis lies in the estimation of the number of segments that requires the definition of appropriate penalty function and constant. We define a new procedure that estimates the number of segments adaptively to the data. This method selects the number of segments with high accuracy compared to previously mapped aberrations, and seems to be more efficient compared to others proposed to date. The use of dynamic programming remains central to localizing the break-points, and the simulation results show that when the good number of segments are selected, the algorithm localizes the break-points very close to the truth. Assessing the number of segments in a model is theoretically complex, and requires the definition of a precise model of inference. To that extent, microarray CGH analysis not only requires computational approaches, but also a careful statistical methodology.

## Methods

### Materials

We briefly present the data we used in this article. The first data we use in the Results Section consist of a single experiment on fibroblast cell lines (Coriell Cell lines) whose chromosomal aberrations have been previously mapped. Those defaults concern partial or whole chromosome aneuploidy. This data have been previously used by other authors [10]. The second group of data used in the Results section is described in [6]. A test genome of Bt474 cell lines is compared to a normal reference male genome. The last data set used is described in [16] and consists of 125 primary colorectal tumors that were surgically dissected and frozen. The arrays used for these analysis are BAC arrays described in [6].

### Models and Likelihoods

In this section, we define the models 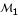 and 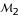. Let us consider a CGH profile, and note *y*_*t*_, the log_2_-ratio of the intensities for the *t*^*th *^BAC on the genome. Precisely *y*_*t *_represents the average signal obtained from the replicated spots on the slide. BACs are the basic units in our model, and are ordered according to their physical position. We suppose that the *y*_*t *_are the realizations of independent random variables {*Y*_*t*_}_*t *= 1...*n*_, with Gaussian distributions 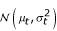. We assume that *K *- 1 changes affect the parameters of the distribution of the *Ys*, at unknown coordinates (*t*_0_, *t*_1_, *t*_2_,...,*t*_*K *- 1_, *t*_*K*_) with convention *t*_0 _= 1 and *t*_*K *_= *n*, and that the parameters of the *Ys *distributions are constant between two changes:


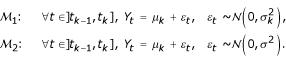


where *μ*_*k *_is the mean of the *k*^*th *^segment. Model 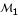 specifies that the variance is segment-specific (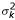), whereas 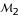 considers that the variance is common between segments (*σ*^2^). Since BACs are supposed to be independent, the log-likelihood can be decomposed into a sum of "local" likelihoods, calculated on each segments: 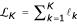, with


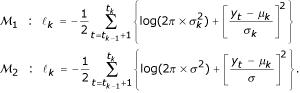


### Estimation of the segment's mean and variance

Given the number of segments *K *and the segments' coordinates *(t*_0_, *t*_1_, *t*_2_,...,*t*_*K*-1_, *t*_*K*_), we estimate the mean and the variance for each segment using maximum likelihood :


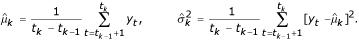


If the variance of the segments is homogeneous, its estimator is given by:


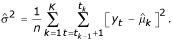


Notice that when the segment coordinates are known, the estimation of the mean and variance for each segment is straightforward. Then, the key problem is to estimate *K *and (*t*_0_, *t*_1_, *t*_2_,...,*t*_*K *- 1_, *t*_*K*_). We will proceed in two steps: in the first step, we will consider that the number of segments is known, and the problem will be to estimate the *t*_*k*_s, that is, to find the best partition of a set of *n *individuals into *K *segments. In the second step, we will estimate the number of segments, using a penalized version of the likelihood.

### A segmentation algorithm when the number of segments is known

When the number of segments *K *is known, the problem is to find the best partition of {1,...,*n*} into *K *segments, according to the likelihood, where *n *is the size of the sample. An exhaustive search becomes impossible for large *K *since the number of partitions of a set with *n *elements into *K *segments is 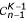. To reduce the computational load, we use a dynamic programming approach (programs are coded in MATLAB language and are available upon request). Let 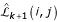 be the maximum log-likelihood obtained by the best partition of the data {*Y*(*i*), *Y*(*i *+ 1),...,*Y*(*j*)} into *k *+ 1 segments, with *k *break-points, and let note 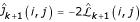. The algorithm is as follows:


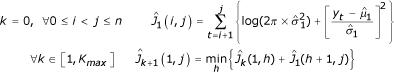


Dynamic programming takes advantage of the additivity of the log-likelihood described above, considering that a partition of the data into *k *+ 1 segments is a union of a partition into *k *segments and a set containing 1 segment. This approach presents two main advantages: it provides an exact solution for the global optimum of the likelihood [17], and reduces the computational load from 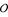
(*n*^*K*^) to 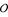
(*n*^2^) for a given *K *(the algorithm only requires the storage of an upper *n *× *n *triangular matrix). At the end of the procedure, the quantities 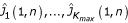 are stored and will be used in the next step. Notice that this problem of partitioning is analogous to the search for the shortest path to travel from one point to another, where 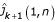 represents the total length of a (*k *+ 1)-step-path connecting the point with coordinate 1 to the point with coordinate *n*.

### An adaptive method to estimate the penalty constant

The purpose of this section is to explain an adaptive method to estimate the number of segments. Further theoretical developments can be found in [14]. If we consider that the likelihood 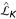 measures the adjustment of a model with *K *segments to the data, we aim at selecting the dimension for which 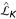 ceases to increase significantly. For this purpose, let us define a decreasing sequence (*β*) such as *β*_0 _= ∞ and


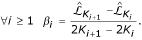


If we represent the curve (*pen*(*K*), 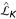), the sequence of *β*_*i *_represents the slopes between points (*pen*(*K*_*i *+ 1_), 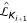) and (*pen*(*K*_*i*_), 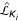), where the subset {(*pen*(*K*_*i*_),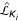),*i *≥ 1}) is the convex hull of the set {(*pen*(*K*),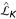)}.

Since we aim at selecting the dimension for which 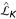 ceases to increase significantly, we look for breaks in the slope of the curve. We define *l*_*i*_, the variation of the slope, that exactly corresponds to the length of the interval ]*β*_*i*_, *β*_*i *- 1_] : *l*_*i *_= *β*_*i *- 1 _- *β*_*i*_. The length of these intervals is directly related to the second derivative of the likelihood. The automatic procedure to estimate the number of segments is then to calculate the second derivative (finite difference) of the likelihood:


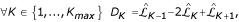


and we select the highest number of segments *K *such that the second derivative is lower than a given threshold :


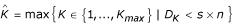


Other procedures have been developed to automatically locate the break in the slope of the likelihood. Nevertheless, the criterion we use can be interpreted geometrically and is easy to implement. The choice of the constant *s *is arbitrary. According to our experience, a threshold *s *= -0.5 seems appropriate for our purpose. A criticism that can be made to this procedure is its dependency on the threshold which is chosen. Nevertheless, it is important to point out that despite this thresholding the procedure remains adaptive, since the penalty constant is estimated according to the data.

### Simulation studies

We performe numerical simulations to assess the sensitivity of our procedure to the addition of noise. In the first case, we simulate 100 points with K* = 5 segments. In the first case Figure 3, the segments are regularly spaced and the difference of the means between two segments is *d *= 1. In the second case (Figure 4) the segments are irregularly spaced and the difference of the means varies between *d *= 2 and *d *= 0.5. The standard deviation of the Gaussian errors varies from *σ *= 0.1 to *σ *= 2. Each configuration is simulated 500 times, and we calculate the average selected number of segments over 500 simulations. In order to assess the performance of the dynamic programming algorithm, we calculate the empirical probability over 500 simulations for a break-point to be located at coordinate *t *(for *t *= 1 to 100).

## Authors' contributions

FP developed the statistical models and the programs dedicated to array CGH data analysis, ML developped the adaptive selection of the number of segments. SR, CV and JJD supervised the study.

## Figures and Tables

**Table 1:** Constants and penalty funtions for different penalized criteria, in a heteroscedastic model with *K *segments.

criterion	*β*	*pen*(*K*)
AIC	I	2*K*
BIG	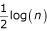	2*K*
Jong (2003)	10/3	3*K *- 1
Lebarbier (2003)	adaptive	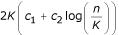
Lavielle (2003)	adaptive	2*K*
